# Efficient Claustrum Segmentation in T2-weighted Neonatal Brain MRI Using Transfer Learning from Adult Scans

**DOI:** 10.1007/s00062-021-01137-8

**Published:** 2022-01-24

**Authors:** Antonia Neubauer, Hongwei Bran Li, Jil Wendt, Benita Schmitz-Koep, Aurore Menegaux, David Schinz, Bjoern Menze, Claus Zimmer, Christian Sorg, Dennis M. Hedderich

**Affiliations:** 1 Department of Diagnostic and Interventional Neuroradiology, Klinikum rechts der Isar, Technical University of Munich, Ismaninger Strasse 22, 81675 Munich, Germany; 2 TUM-NIC Neuroimaging Center, Munich, Germany; 3 Department of Informatics, Technical University of Munich, Munich, Germany; 4 Department of Quantitative Biomedicine, University of Zurich, Zurich, Switzerland; 5 Department of Psychiatry, Klinikum rechts der Isar, Technical University of Munich, Munich, Germany

**Keywords:** Claustrum, Newborn infants, Deep learning, Image segmentation, Transfer learning

## Abstract

**Purpose:**

Intrauterine claustrum and subplate neuron development have been suggested to overlap. As premature birth typically impairs subplate neuron development, neonatal claustrum might indicate a specific prematurity impact; however, claustrum identification usually relies on expert knowledge due to its intricate structure. We established automated claustrum segmentation in newborns.

**Methods:**

We applied a deep learning-based algorithm for segmenting the claustrum in 558 T2-weighted neonatal brain MRI of the developing Human Connectome Project (dHCP) with transfer learning from claustrum segmentation in T1-weighted scans of adults. The model was trained and evaluated on 30 manual bilateral claustrum annotations in neonates.

**Results:**

With only 20 annotated scans, the model yielded median volumetric similarity, robust Hausdorff distance and Dice score of 95.9%, 1.12 mm and 80.0%, respectively, representing an excellent agreement between the automatic and manual segmentations. In comparison with interrater reliability, the model achieved significantly superior volumetric similarity (*p* = 0.047) and Dice score (*p* < 0.005) indicating stable high-quality performance. Furthermore, the effectiveness of the transfer learning technique was demonstrated in comparison with nontransfer learning. The model can achieve satisfactory segmentation with only 12 annotated scans. Finally, the model’s applicability was verified on 528 scans and revealed reliable segmentations in 97.4%.

**Conclusion:**

The developed fast and accurate automated segmentation has great potential in large-scale study cohorts and to facilitate MRI-based connectome research of the neonatal claustrum. The easy to use models and codes are made publicly available.

**Supplementary Information:**

The online version of this article (10.1007/s00062-021-01137-8) contains supplementary material, which is available to authorized users.

## Introduction

The claustrum is a thin and sheet-like gray matter structure of the mammalian forebrain between the striatum and insular cortex, or more precisely, in humans between the external and extreme capsule [1, 2]. Examining the claustrum is challenging due to its small size, ambiguous shape, and deep brain location. The function of the claustrum remains unclear, and most investigations are based on animal studies, which highlights the need for imaging-based studies in humans. Preliminary findings suggest that the claustrum is relevant for consciousness [3], task switching, salience network organization, attention guiding, and top-down control [4–8]. Human studies suggest a role of the claustrum in selective attention and task switching [9]; however, these investigations are usually limited to small sample sizes [10, 11]. In large cohorts, common manual claustrum segmentation would be very laborious and time consuming.

Moreover, there is a lack of knowledge about claustrum development in humans. Most studies focus on animals, while macrostructural and microstructural maturation in humans remain unknown [1, 12, 13]. It has been shown that there are significant differences between very preterm and term-born young adults in patterns of BOLD activity in clusters centered on the claustrum during a learning task [14]. A clear rationale to study claustrum development, particularly in premature-born neonates, comes from its shared ontogenetic trajectory with so-called subplate neurons [15]. The subplate neurons are a predominantly transient cell population and are therefore vulnerable to hypoxic-ischemic events and thus, play a key pathophysiologic role for disturbed neurodevelopment after premature birth [16–21]. This is underlined by a previous study showing altered claustrum microstructure in premature-born adults [22], which is a finding with potentially significant implications. Examination of the claustrum and altered claustrum structure in neurodevelopmental disorders such as impaired development after premature birth may lead to the establishment of imaging biomarkers for subplate neuron pathology. This may also be extended to other neurodevelopmental disorders with presumed subplate neuron pathology, such as schizophrenia and autism spectrum disorder [23]. Hence, close examination and characterization of claustrum development in younger cohorts is of special interest; however, data about the claustrum in a sizable neonatal cohort are missing, mostly due to the lack of adequate automated segmentation methods.

Recently, automated segmentation of the human claustrum in adults has been investigated by structural approximation to the dorsal claustrum [24] and a two-dimensional deep-learning approach [25]. Furthermore, a multiview deep learning-based model has been proposed [26] to segment the human claustrum trained on a large annotated dataset; however, no reliable automated segmentation method for the claustrum in neonatal MRI exists.

To fill this gap, this study presents an efficient deep learning-based segmentation framework using manual expert annotations of the claustrum in a sophisticated cohort of neonatal MRI from the developing Human Connectome Project (dHCP) [27] comprising ongoing brain development. Transfer learning [28] enabled reuse of available artificial intelligence models despite different neuroanatomy, scanner, image sequence, and image resolution shift, and drastically shortened the training time to 90 min. Segmentation accuracy was evaluated based on three canonical performance metrics, volumetric similarity (VS), 95th percentile of the Hausdorff distance (HD95), and Dice similarity coefficient (DSC), and compared with intrarater and interrater reliability of manual segmentation. The proposed technique was also compared to a nontransfer learning approach. The study provides an insight into the training process by quantifying the amount of manually annotated images needed for good segmentation results. Lastly, the deep learning model was applied to the whole, large-scale dHCP dataset to see how its output holds out against rigorous visual quality control. An accuracy drop in young neonates was analyzed and solved by an age-stratified training set. Training and testing code and models are released on GitHub for other research groups. A detailed claustrum segmentation protocol is in the Online Supplement. In parallel, the proposed transfer learning approach serves as a template for similar segmentation tasks of intricate and small structures in the developing brain.

## Material and Methods

In the following parts, the single term “model” refers to a 2D artificial neural network while “combined model” integrates several 2D models (*see* Section Multiview Convolutional Neural Network). Whereas manually acquired tracing of the claustrum is always described with the term “manual segmentation”, the output of a model is described as “automated segmentation” or “prediction” in an interchangeable way.

**Fig. 1:**
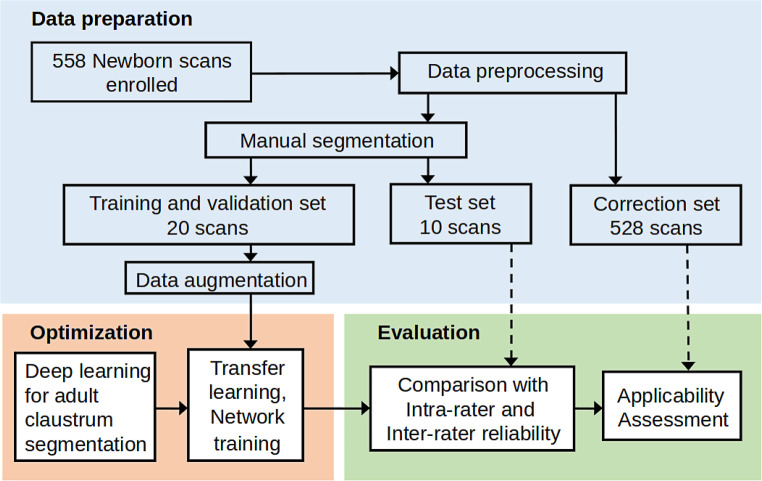
A schematic view of the image segmentation and evaluation pipeline of this study. It includes three stages: 1) data preparation, 2) model optimization and 3) framework evaluation

The general image processing diagram in this work includes three stages shown in Fig. 1. Data preparation deals with the enrolment of 558 subjects, image preprocessing and manual segmentation of neonate claustrum. Optimization aims to perform transfer learning and train a deep-learning model with the manual segmentations provided in the first stage. Finally, the evaluation investigates the effectiveness and the applicability of the established model on unseen data including failure analysis and model improvement. The following two sections describe the datasets and evaluation metrics in this study.

### Datasets

All 558 three-dimensional MRI scans of newborns from the second data release of the developing Human Connectome Project (dHCP)^1^ were included. The large-scale public dataset contains 558 brain MRI of 505 neonates from 23 to 44 weeks postconceptional age with a mean (± standard deviation) scan age of 40 (± 3) gestational weeks. In detail, the study comprises 378 scans of term-born neonates and 180 scans of preterm-born neonates, including 82 scans of very preterm-born neonates (birth age < 32 gestational weeks). Data involve previously known at risk groups for neurodevelopmental disorders and incidental findings in clinically unsuspicious neonates [29, 30]. The explicit inclusion and exclusion criteria are shown on the dHCP website^2^. Recruitment and scanning took place at the Evelina Newborn Imaging Centre, St Thomas’ Hospital in London, UK [29]. Written consent by the parents was previously requested [27]. Due to immature structures with different tissue composition than in adults, the preferred structural image sequence in neonatal brain MRI are T2-weighted (T2-w) scans. Thus, the dHCP favored this sequence in data preprocessing steps [29] and we focused on it for our study. Images were acquired with a 3T Philips Achieva with a repetition time TR = 12,000 ms and echo time TE = 156 ms, isotropic reconstructed voxel size of 0.5 mm and scanning in axial (SENSE factor: 2.11) and sagittal (SENSE factor: 2.60) plane with a neonatal 32 channel head coil [27]. The structural brain images passed visual quality control, brain extraction, and were preprocessed by retrospective motion and bias correction by the dHCP [29, 31].

Out of this dataset, 30 randomly chosen subjects passed manual segmentation. Subsequently, these scans were split in a training set of 20 subjects and a test set comprising 10 scans for evaluation. The remaining 528 scans served as correction set and did not undergo manual segmentation. Training, test, and correction sets are consistent throughout the experiments (Table 1).

**Table 1:** Characteristics of the dataset in this study. The dataset consists of 558 subjects from the developing Human Connectome Project. For each dataset, the count of scans and the mean scan age (range) in gestational weeks are given

Scanner	Field strength	Voxel size (mm^3^)	Training set; scan age	Test set; scan age	Correction set; scan age
Philips Achieva (Philips, Best, The Netherlands)	3T	0.5 × 0.5 × 0.5	20 scans 39.9 (36.1–42.6)	10 scans 40.4 (38.7–42.3)	528 scans 40.0 (29.3–45.1)

The manual segmentation was performed with ITK-SNAP-v3.6.0^777^ [32] on a Wacom Intuos M tablet (Wacom, Kazo, Saitama, Japan). The first rater was under close supervision of a board-certified neuroradiologist with 10 years of experience including imaging for a neonatal intensive care unit and 5 years of experience pertaining to imaging of premature-born individuals and related outcomes. The detailed segmentation protocol, which assures a constant structure for more objective and stable results, is described in the Online Supplement. Despite this approach, it remains challenging to define the exact boundaries of the small claustrum due to the ambiguity. To quantify the intrarater reliability of manual segmentation, the first rater traced the right and left claustrum of the 10 subjects in the test set at two time points. Furthermore, these 10 subjects were manually segmented by a second rater with the same protocol to assess interrater reliability.

### Model Evaluation

Given a manual segmentation mask *M* and a predicted segmentation mask *P*, three different evaluation metrics assessed the model performance:

#### Volumetric Similarity (VS)

While *V*_*M*_ and *V*_*P*_ are the volumes of the claustrum in *M* and *P, *respectively, the volumetric similarity (VS) between them is defined as:

$$VS\left[\% \right]=1-\frac{| V_{M}-V_{P}| }{| V_{M}+V_{P}| }$$

#### 95th Percentile of the Hausdorff Distance (HD95)

The Hausdorff distance (HD) is a common score to measure the surface distance between two masks *M* and *P* [33]:

$$HD\left(M,P\right)=\max \left\{\underset{x\in M}{\sup }\underset{y\in P}{\inf }d\left(x,y\right),\underset{y\in P}{\sup }\underset{x\in M}{\inf }d\left(x,y\right)\right\}$$

*d*(*x,y*) denotes the Euclidean distance of x and y, *sup* terms the supremum and *inf* the infimum. We used the 95th percentile instead of the maximum (100th percentile) distance to discount single outliers.

#### Dice Similarity Coefficient (DSC)

$$DSC=\frac{2\left(M\cap P\right)}{| M| +| P| }$$

The Dice similarity coefficient (DSC) quantifies the spatial overlap between manual segmentation *M* and prediction mask *P*.

#### Evaluation Protocol

##### K-fold Cross-validation

The model’s overall performance was evaluated with k‑fold cross-validation with 20 subjects in the training/validation set. While *k* was set to 5, in each split 80% of the scans were pooled into the training set and the remaining 20% were used for validation. After five iterations, all subjects were evaluated in the validation phase.

##### Evaluation on a Test Set

The model was optimized on 20 subjects. The combined model was evaluated on a test set with 10 subjects and compared with intrarater and interrater reliability.

##### Applicability Assessment

The combined model was applied to the correction set with 528 subjects. These predictions were compared with their subsequently manually corrected correlates.

### Additional Preprocessing and Postprocessing

#### Image Preprocessing

We performed additional steps on top of the basic preprocessing steps carried out by dHCP protocol (Sect. Datasets). First, a *z-*score normalization standardized the brain voxel intensities for each scan as proposed in [26]. Second, every slice was cropped to a uniform size of 200 × 200 pixels to exclude background information. Third, the first and last 25% of the slices were removed based on empirical decision to focus on central parts of the brain, which include the claustrum, and to lower the computational time.

#### Segmentation Postprocessing

After generating a segmentation, two postprocessing steps were applied to it: 1) the segmentation maps were padded with respect to the original size, i.e., an inverse operation to the previous second preprocessing step and 2) an according sequence to preprocessing step three to remove some artifacts.

### Data Augmentation

In contrast to expensive manual segmentation, data augmentation (DA) is a method to enlarge the amount and the diversity of training data. A stack of selective transformations, including moderate shift, scaling, rotation, and shearing to the image slices and the corresponding masks, resulted in doubled training data (*see* Fig. S1 in the Online Supplement for selection of DA methods). For comparison, the same models were trained with and without DA and their performance was assessed on the validation set. There was no significant difference regarding the VS; however, DA led to a significant improvement of automated segmentation concerning HD95 and DSC (see Table S1). For the stated reasons, data augmentation enriched the following experiments.

### Multiview Convolutional Neural Network

As automated neonatal claustrum segmentation is not feasible to conventional atlas-based methods, we adopted a supervised deep-learning approach developed for adults [26]. While training, the model takes labeled slices of MR images as input data and adapts its parameters towards accurate prediction by minimizing the loss function (Sect. Parameter Setting and Computation Complexity). Finally, the trained model can be applied to trace the claustrum in unseen neonatal images. Based on the beneficial multiview approach proposed in [26], we train coronal and axial deep convolutional neural networks on 2D single-view slices after parsing 3D MRI volume into axial and coronal views. In the test stage, the predictions are automatically combined on a voxel-wise level.

The network architecture of the convolutional neural network [26] adapted to the neonatal image format is shown in Fig. S2. It has a U-shape [34] with a down-convolutional part that extracts features of the T2‑w input scans. The up-convolutional part assigns the categories claustrum or non-claustrum to each pixel conforming a segmentation of the claustrum.

### Transfer Learning

**Fig. 2:**
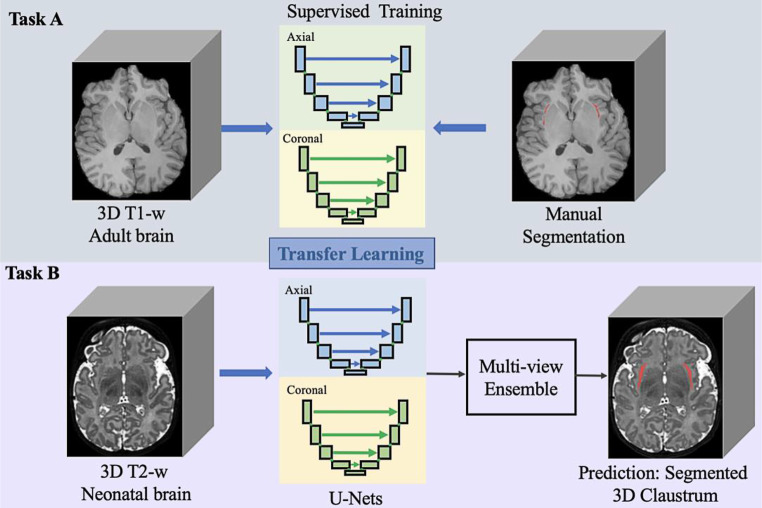
A schematic view of the proposed segmentation method using transfer learning and multiview convolutional neural networks to segment the newborn claustrum given limited data. The network for each view (i.e., axial and coronal) is a 2D convolutional network architecture, and it takes the raw images as the input and predicts the claustrum segmentation

Transfer learning is typically performed using a designed model and pretrained weights from one source task and fine-tuning on the target task. In this work, the knowledge from task A: human claustrum segmentation in T1‑w adult images, was transferred to task B**:** claustrum segmentation in high-resolution T2‑w images of neonates scanned in a range of 21 gestational weeks with ongoing brain development. As shown in Fig. 2, we used the same model and directly took its weights learned from task A. Then the multiview networks were optimized with only 20 T2‑w scans with manual segmentations for task B. It took around 90 min for the whole training process and 6s for automated segmentation using a common NVIDIA (Santa Clara, CA, USA) graphics processing unit (GPU). The high efficiency of our framework is explained in the following sections.

### Parameter Setting and Computation Complexity

The hyperparameters were chosen consistently for all experiments and optimized efficiency and accuracy. Each model was trained for 30 epochs to avoid overfitting and to keep a low computational cost by monitoring VS and DSC on a validation set. The batch size was empirically set to 60 as a relatively large batch size tended to a more stable training than a smaller batch size mainly due to the imbalanced nature of the training set. The learning rate was set to 0.0002. Non-TL models, which were prepared for comparison reasons, were trained for 275 epochs (see Fig. S5). The other hyperparameters were similar as for TL.

In the claustrum segmentation task, the distribution of claustrum voxels and non-claustrum voxels are highly imbalanced. To handle this issue, the Dice loss was used as a loss function to minimize the difference between manual segmentation and prediction during training [26, 35, 36].

All experiments were performed on a Linux workstation running Ubuntu 20.04 (Canonical Ltd., London, UK), with 64 GB RAM. The number of trainable parameters in the single-view architecture is 2,494,529. The model was trained on one NVIDIA Titan-Xp GPU with 12 GB of GDDR5X memory. Training a single model for 30 epochs on a training set containing 4200 images with a size of 200 × 200 pixels took only around 12 min. For model robustness, three axial view models and three coronal view models were trained and aggregated at a voxel-wise level resulting in a combined model. Predicting the segmentation of one scan with 192 slices by such a combined model took around 90 s using an Intel (Santa Clara, CA, USA) Xeon central processing unit (CPU) (E3-1225v3) and only 6 s when using a GPU.

## Results

### Segmentation Accuracy

**Fig. 3:**
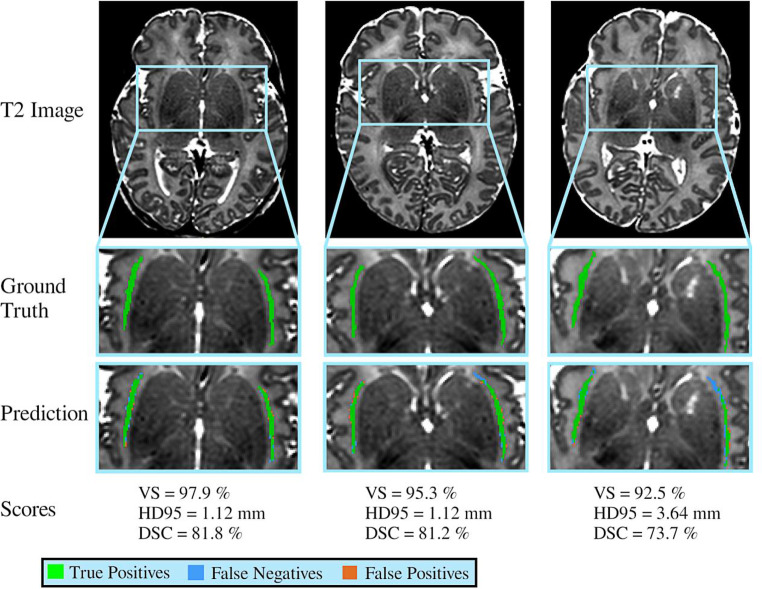
Segmentation results of three sample cases. In the automated segmentation masks, the green pixels represent true positives, the blue ones represent false negatives, and orange ones represent false positives. Examples are sorted according to accuracy as determined by the Dice similarity coefficient (DSC). *VS* volumetric similarity, *HD95* 95th percentile of Hausdorff distance

Three examples of automated claustrum segmentation are shown in Fig. 3.

**Fig. 4:**
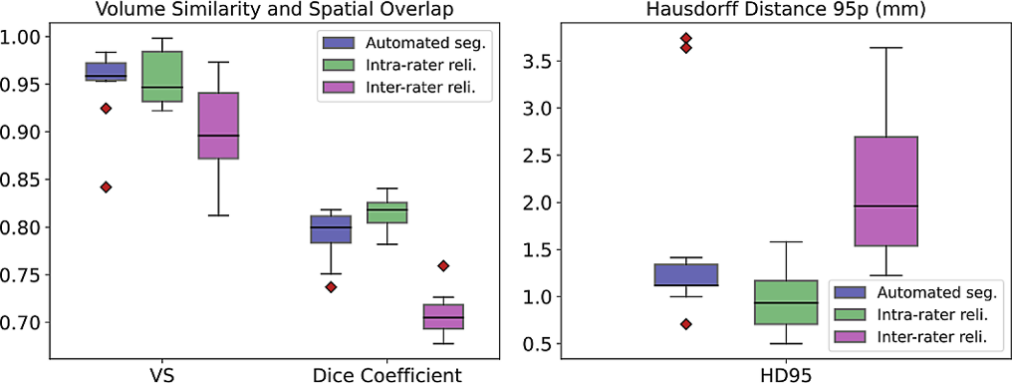
Segmentation performance of the proposed method on the test set (automated seg.) and comparison to intrarater and interrater reliability (reli.). In comparison with intrarater reliability, automated segmentation is significantly inferior concerning the 95th percentile of the Hausdorff distance (HD95) and Dice coefficient. In comparison with interrater reliability, automated segmentation is significantly superior regarding volumetric similarity (VS) and Dice coefficient (in arbitrary unit, respectively)

To assess the accuracy of our combined model for automated claustrum segmentation, we calculated three performance metrics, volumetric similarity (VS), 95th percentile of the Hausdorff distance (HD95), and Dice similarity coefficient (DSC), on the test set and compared its performance with intrarater and interrater reliabilities on the same set (for detailed results see Table 2 and Fig. 4). The proposed method yielded median VS, HD95, and DSC of 95.9%, 1.12 mm, and 80.0%, respectively. Repeated segmentation by the same reader led to median VS, HD95, and DSC of 94.6%, 0.93 mm, and 81.8%, respectively and is referred to as intrarater reliability. Segmentation of the test set by both readers 1 and 2 led to median VS, HD, and DSC of 89.6%, 1.96 mm, and 70.5%, respectively and serves as interrater reliability. Comparing the automated segmentation with intrarater reliability with a Wilcoxon signed-rank test, we found significantly lower HD95 (*p* = 0.011) and higher DSC (*p* < 0.005) for repeated manual segmentation by the same reader. Comparing the automated segmentation with interrater reliability with the same statistical test, the automated segmentation algorithm achieved significantly higher VS (*p* = 0.047) and higher DSC (*p* < 0.005). These results show that the accuracy of our automated segmentation approach is comparable to intrarater reliability with minimally inferior results at HD95 and DSC and that it is superior to interrater reliability in two out of three performance metrics.

**Table 2:** Performance comparison between the accuracy of the automated segmentation achieved by the combined model and the intrarater reliability or interrater reliability, respectively. ↓ indicates that a smaller value represents better performance; *bold* *p*-values are significant (p≤0.05)

Metric, median (IQR)	Automated segmentation (AS)	Intrarater reliability	Interrater reliability	*p*-value (AS vs. intrarater)	*p*-value (AS vs. interrater)
VS, in %	95.9 (95.4, 97.2)	94.6 (93.2, 98.4)	89.6 (87.2, 94.1)	0.959	**0.047**
HD95, in mm↓	1.12 (1.12, 1.34)	0.93 (0.71, 1.17)	1.96 (1.54, 2.69)	**0.011**	0.203
DSC, in %	80.0 (78.4, 81.2)	81.8 (80.4, 82.6)	70.5 (69.3, 71.8)	**<** **0.005**	**<** **0.005**

*VS* volumetric similarity, *HD95* 95th percentile of Hausdorff distance, *DSC* Dice similarity coefficient, *IQR* interquartile range

### Efficiency of Transfer Learning in Comparison with Nontransfer Learning

To evaluate the efficiency of the transfer learning technique (TL), we compared it with the vanilla approach, i.e., training from scratch (non-TL). Internal fivefold cross-validation on the training set was performed with both methods. VS, HD95, DSC and training times were recorded and compared (for details, see Table S2 and Fig. S6 in the Online Supplement). The TL method achieved a median VS, HD95, and DSC of 95.3%, 1.06 mm, and 78.9%, respectively, and training took around 90 min. The non-TL approach led to a median VS, HD95, and DSC of 93.5%, 1.00 mm, and 79.4%, respectively, and a training time of 17.5 h. Comparing these results with a Wilcoxon signed-rank test, the TL method showed a significantly superior VS (*p* = 0.050), inferior HD95 (*p* = 0.016), and no significant difference regarding DSC (*p* = 0.452). Concerning the time needed for training, TL was more than 11 times faster than training from scratch. This finding suggests that TL and non-TL achieve comparable performance but TL is far more time efficient.

###  Data Range Needed for Transfer Learning

**Fig. 5:**
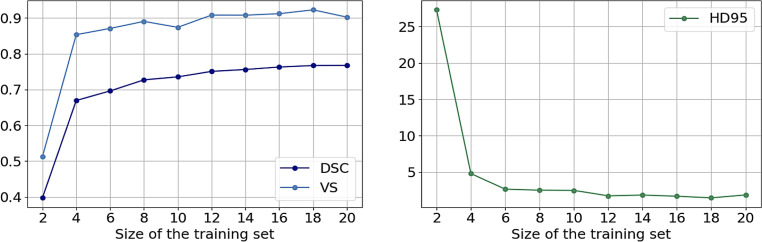
The left diagram shows volumetric similarity (VS) and Dice similarity coefficient (DSC), both in arbitrary unit, of the test set of models trained with different amounts of training data (measured in scans). The right graph presents the 95th percentile of Hausdorff distance (HD95) in mm of these models. The performance mainly increases till around 12 images in the training set and saturates afterward

To determine how much training data are needed for transfer learning, a model was trained with various training set sizes, i.e., the first model was trained with two scans and the training set was gradually increased with two scans for the following models. The VS, HD95, and DSC were determined on the test set. The model performance improved with increasing training set up to 12 images (Fig. 5). Beyond this size, there only remained a minimal shift of DSC up to 18 images. Surprisingly, even a training set of four scans can reach relatively high scores. This result indicates that transfer learning can deal effectively with a small training set of around 12 scans and their corresponding manual segmentations. Additional results of how much data are needed for non-transfer learning are shown in Fig. S6 in the Online Supplement.

### Applicability Assessment on a Large-scale Held-out Correction Set

**Fig. 6:**
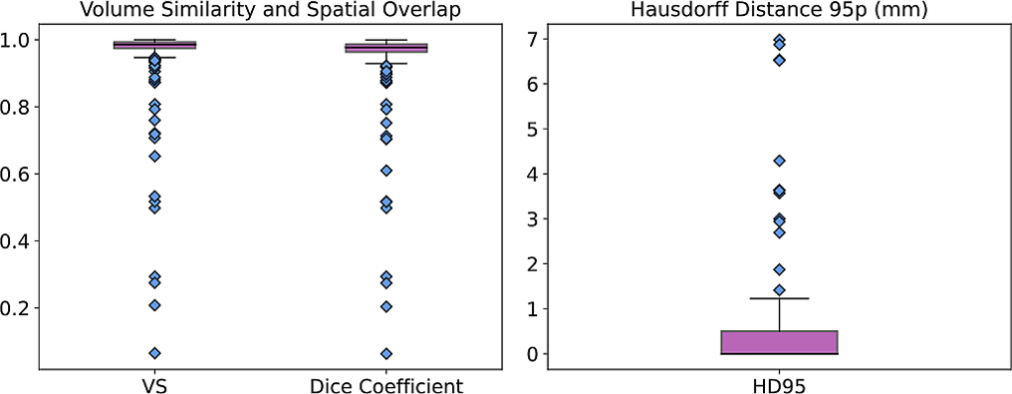
Volumetric similarity (VS, in arbitrary unit), Dice similarity coefficient (DSC, in arbitrary unit) and 95th percentile of the Hausdorff distance (HD95, in mm) of 528 automated segmentations of the claustrum. Except for several outliers with medium or low accuracy, the majority shows high performance in all three metrics within a small range

To test the applicability of the proposed deep-learning-based approach, the model predicted the claustrum in the held-out correction set of 528 scans. Subsequently, we corrected the predictions manually where needed and compared predicted and corrected segmentation by charging VS, HD95, and DSC. The median VS, HD95, and DSC were 98.5%, 0.00 mm, and 97.7% (see Fig. 6), respectively. In total, we found 14 scans of which the DSC of the claustrum segmentation was less than the mean intrarater reliability of 81.8%, corresponding to 2.7% of the whole correction set. In three of these scans, the right claustrum was not detected at all. These subjects, two female and one male neonate, were born in a range of gestational age 26.1–28.7 weeks and scanned between 29.3 and 31 gestational weeks, suggesting an unfavorable impact of very young age on the accuracy of the prediction. A performance comparison between the right and left claustrum is shown in the Online Supplement in Fig. S8 and Table S3.

**Fig. 7:**
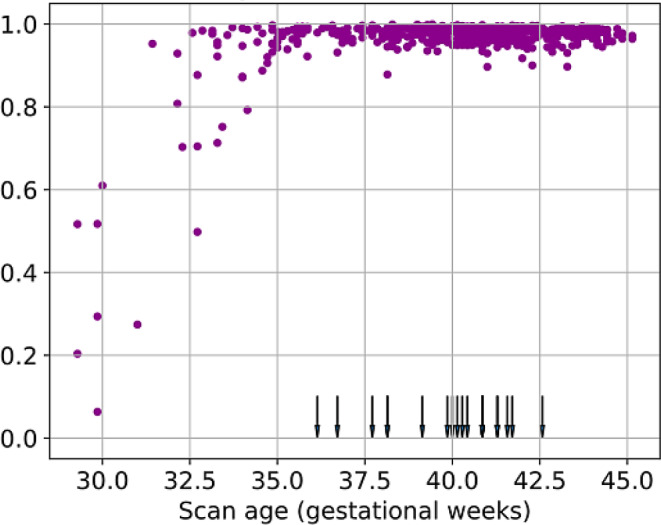
Dice similarity coefficient (DSC, in arbitrary unit) of 528 manually corrected and initial automated segmentations of right and left claustrum depending on the scan age. The head-down arrows indicate the scan age of the training subjects. Subjects with relatively low segmentation performance are younger than the training samples

In a further analysis, we tried to explain the result of the outliers with low performance (DSC < 81.8%). As shown in Fig. 7, all predictions with low accuracy were obtained in newborns before 35.0 gestational weeks. In subjects older than 35.0 gestational weeks, the combined model reached a high accuracy (DSC > 81.8%) in 100% of the scans. Notably, the training subjects were scanned in a range of 36.1–42.6 gestational weeks which presents a domain shift compared to the correction set. Three exemplary young subjects are presented in Fig. S9 in the Online Supplement. This indicates an age-dependent artificial intelligence performance which could be attributed to restricted training samples. Thus, an adjustment of the training samples should improve the performance in young subjects. To test this hypothesis, we replaced two older neonates (scan age around 40 gestational weeks) by two very preterm-born subjects (scan age around 29 gestational weeks) to obtain age stratification in the training set. This led to significantly higher performance in a group of the five young neonates (scan age 29.3–32.7 gestational weeks) with the lowest DSC in Fig. 7 (see Fig. S10) and, surprisingly, also in the original test set (scan age 38.7–42.3 gestational weeks) (see Table S4). To sum up, a scan age stratification of the training set globally improved the model in this developing cohort.

## Discussion

This study demonstrated that fully automated claustrum segmentation in T2-weighted neonatal brain MRI is feasible by using deep learning. While the gray matter structure is too small for atlas-based labeling and too intensive for large-scale manual labeling, we successfully implemented a transfer learning (TL) approach building on a previous method for claustrum segmentation in adult brain MRI, leading to segmentation accuracy comparable to intrarater reliability and superior to interrater reliability. The released models and codes will facilitate MRI-based research of the newborn claustrum through automated segmentation. In addition, the presented approach can function as a template for automated segmentation of other intricate structures in the developing neonatal brain or transfer learning to different datasets by published model training and testing code.

The proposed transfer-learning-based method offers high segmentation accuracy. A transfer learning approach fits to our segmentation problem in neonates because DL-based segmentation approaches are more common in adults but not in neonates e.g., amygdala nuclei or hypothalamus [37, 38]. In principle, evidence for the possibility to transfer adult segmentation of specific subcortical regions to neonates was demonstrated. The performance of our segmentation approach was evaluated with three metrics, volumetric similarity (VS), 95th percentile of the Hausdorff distance (HD95) and the Dice similarity coefficient (DSC), on a test set and compared with intrarater and interrater reliability of the same test set. Automated segmentation was partly inferior to intrarater reliability but significantly superior to interrater reliability concerning two scores. In comparison with the prior study of automated adult claustrum segmentation [26], all scores of the neonate claustrum were improved. A possible explanation for this might be the enhanced resolution of newborn MRI/adult MRI of 0.5/1.0 mm isotropic voxel size suggesting that a higher image resolution and a larger volume in voxels lead to higher accuracy. The overall performance level is lower than in comprehensive white or gray matter segmentation reaching a Dice score of about 95% [39]; however, the accuracy accords with observations in other ambiguous and small structures like the hypothalamus and its subnuclei with a Dice score of 51–84% [37]. Altogether, the deep learning method deals with the delicate and variable neonatal claustrum despite a short training set of 20 scans segmented by one rater and outperforms the variability of several human raters, which is especially relevant in large datasets.

When matching TL with non-TL, both options had comparable performance but TL was more time efficient. The methods were optimized individually regarding the number of epochs for training. A second analysis (shown in the Online Supplement) compared the methods with different sizes of the training set with a similar result for larger training sets. With these approaches, a general superiority of TL in terms of our metrics was not certifiable which is consistent with other image segmentation tasks [33]. In the training process, the loss was lower with TL than with non-TL (see Fig. S4 in the Online Supplement) which could be explained by the fact that the Dice loss is not simply confined to the DSC but also represents the certainty of the prediction. To conclude, TL is more time efficient and energy saving than non-TL with stable performance.

We further found that 12 scans for training can be enough to achieve a high model performance. A larger training set hardly improved the accuracy determined with VS, HD95, and DSC. Compared to our previous study, the needed data are much smaller in this neonate project than for adult claustrum segmentation, even after correcting for different image resolutions [26]. Surprisingly, overfitting did not prevent the learning process with small training sets. This could be due to the variability of the images as they come from different layers of the brain. The effect of data augmentation was excluded by testing how much data are needed for models trained without DA. This approach requires more training data for the same performance. We did not test non-DA-non-TL models which would be the exact correlate to the previous adult study. In a large cohort like the dHCP, automated segmentation by deep learning can reduce manual segmentation for the most part as the training and test set are only a small fraction of the whole dataset.

On the question of model applicability, the combined model, an ensemble of three axial and three coronal networks, detected the claustrum correctly in 97.4% of a large held-out correction set. The automated segmentation was compared with manually corrected versions of these predictions and evaluated with VS, HD95, and DSC. The mostly uniform Hausdorff distance of 0.0 mm or 0.5 mm could be attributed to the 95th percentile of this score in conjunction with barely significant adaptations of the predictions. All inadequate predictions with DSC lower than median intrarater reliability were obtained in newborns younger than 35.0 gestational weeks. This result could be explained by the training set which exclusively covered older neonates. Extremely immature neuroanatomy, such as less gyrification or different contrast appearance in MRI than in older neonates, might have distracted our model and resulted in undersegmentation (i.e., false negatives). An age-stratified training set improved the performance in these young subjects and in older neonates. Overall, annotation correction is far more time efficient than manual segmentation from scratch. An automatic selection of subjects that should pass visual control, e.g., due to young age or insufficient detected claustrum volume, could speed up this process further as segmentation in older subjects worked without big mistakes. Consequently, manual correction might be expendable in the latter group. The proposed TL method successfully segments the claustrum with little need for control and correction and enables claustrum analyses in large neonatal cohorts. This facilitates the investigation of the claustrum development and its relation to premature birth. Further investigations are needed to examine the association with other neurodevelopmental disorders, such as schizophrenia and autism spectrum disorders [7].

Despite efficient and accurate automated segmentation, our study has some limitations. First, it is a challenge to precisely define the boundaries of the small and intricate claustrum. Although the dHCP provides a very high isotropic resolution of 0.5 mm and a segmentation protocol structured the process (Online Supplement), the manual segmentation is not perfect because the boundary of specific regions is often ambiguous and its segmentation partly remains subjective, i.e., depends on the rater [37, 40]. This kind of data uncertainty commonly exists in medical image segmentation tasks. One potential solution is to quantify the segmentation uncertainty (e.g., interrater reliability) when building the segmentation model and take the uncertainty of the outcome into account for the downstream analysis (Sect. Segmentation Accuracy). Second, all training images were segmented by one rater. This improves the uniformity of segmentations but could also lead to a bias of the model. Further analyses with two or more raters would be necessary to appraise this impact. Third, the model training was limited to a small dataset that did not cover the whole age range of the dHCP or all neonatal stages of development, which presumably dropped the accuracy, especially in early premature newborns. The model still facilitates manual work in the affected subjects but a strong visual control is important.

In conclusion, this study presented a deep learning approach for automated claustrum segmentation in human neonatal brain MRI. We evaluated the accuracy, compared transfer and non-transfer learning, analyzed how much data are needed for transfer learning and assessed the applicability of the proposed method including a model enhancement by age-stratified training. We conclude that 1) transfer learning is a bit inferior to intrarater reliability but superior to interrater reliability, 2) transfer learning shows similar performance to non-transfer learning and is more time efficient, 3) the prediction accuracy stabilizes with a training set above 12 scans and 4) the combined model applies to a large cohort with predominantly accurate results. The implementation codes are available on *GitHub* to the research community.

## Supplementary Information

The Online Supplement includes a scheme of the deep learning model, statistics of the model training and steps for performance improvement. Findings of the applicability assessment, separated for right and left claustrum, are provided. Additionally, examples of brain images with low model performance are listed together with detailed results of the model trained with an age-stratified training set. Finally, the claustrum segmentation protocol for neonatal brain MRI is presented.

## Abbreviations

AS: Automated segmentation
CPU: Central processing unit
DA: Data augmentation
dHCP: Developing Human Connectome Project
DSC: Dice similarity coefficient
GA: Gestational age
GPU: Graphics processing unit
HD95: 95th percentile of the Hausdorff distance
IQR: Interquartile range
MRI: Magnetic resonance imaging
non-TL: Nontransfer learning
T2‑w: T2-weighted
TL: Transfer learning
VS: Volumetric similarity

## Footnotes

1 http://www.developingconnectome.org/.

2 http://www.developingconnectome.org/study-inclusion-and-exclusion-criteria/.

777 http://www.itksnap.org.
