# Supplementary material for: Efficient Claustrum Segmentation in T2-weighted Neonatal Brain MRI Using Transfer Learning from Adult Scans
Source: Clin Neuroradiol. 2022 Jan 24;32(3):665–76. doi: 10.1007/s00062-021-01137-8 (PMC9424135; doi:10.1007/s00062-021-01137-8)
Supplement: Supplementary file 1 — The Online Supplement includes a scheme of the deep learning model, statistics of the model training and steps for performance improvement. Findings of the applicability assessment, separated for right and left claustrum, are provided. Additionally, examples of brain images with low model performance are listed together with detailed results of the model trained with an age-stratified training set. Finally, the claustrum segmentation protocol for neonatal brain MRI is presented. [file 62_2021_1137_MOESM1_ESM.docx]

**Supplementary Information**

**Title:**

Efficient claustrum segmentation in T2-weighted neonatal brain MRI using transfer learning from adult scans

**Journal:**

Clinical Neuroradiology

**Structure of the Supplement:**

- Details of the training and evaluation process of the deep learning models
- Claustrum segmentation protocol for neonatal brain MRI

**Details of the training and evaluation process of the deep learning models**

**
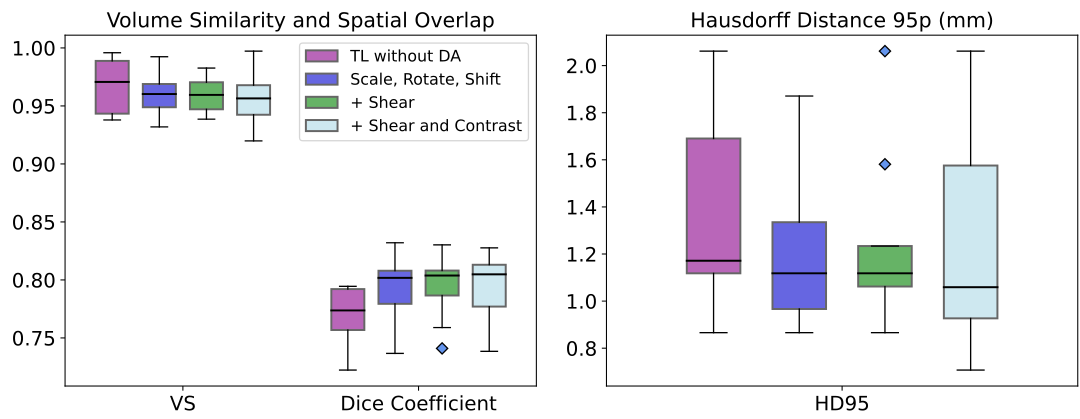
**

**Fig. S1** Performance of models trained, first, without data augmentation, second, with scaling, rotation, and shift as augmentation methods, third, with the latter three methods plus shearing, and fourth, all mentioned methods plus contrast change. For this analysis, one coronal and one axial five-fold cross-validation model were trained for 50 epochs, respectively, and tested on the corresponding validation set. For further application, scaling, rotation, shift, and shearing seemed superior because of the high Dice coefficient, stable volume similarity and low interquartile range of the Hausdorff distance. Additionally, the computational cost was lower than with contrast change. VS=volumetric similarity, HD95=95th percentile of the Hausdorff Distance

**Table S1** Effectiveness of data augmentation. The performance of five-fold cross-validation models with and without data augmentation (DA) was compared. The number of epochs for the training of the models was determined by saturation of the scores of interest: VS=volumetric similarity, HD95=95th percentile of the Hausdorff Distance, DSC=Dice similarity coefficient. DA led to a significant improvement of HD95 and DSC tested with a Wilcoxon signed-rank test. (IQR=Interquartile range)

| Metrics | VS (%)  Median, [IQR] | HD95(mm)↓  Median, [IQR] | DSC (%)  Median, [IQR] | Epochs |
| --- | --- | --- | --- | --- |
| Non-DA | 94.6, [89.0, 98.3] | 1.12, [0.87, 1.58] | 76.1, [72.2, 79.2] | 50 |
| With DA | 95.3, [92.8, 97.8] | 1.06, [0.87, 1.52] | 78.9, [75.4, 80.8] | 30 |
| p-value | 0.132 | **0.007** | **<0.0001** | -- |

**
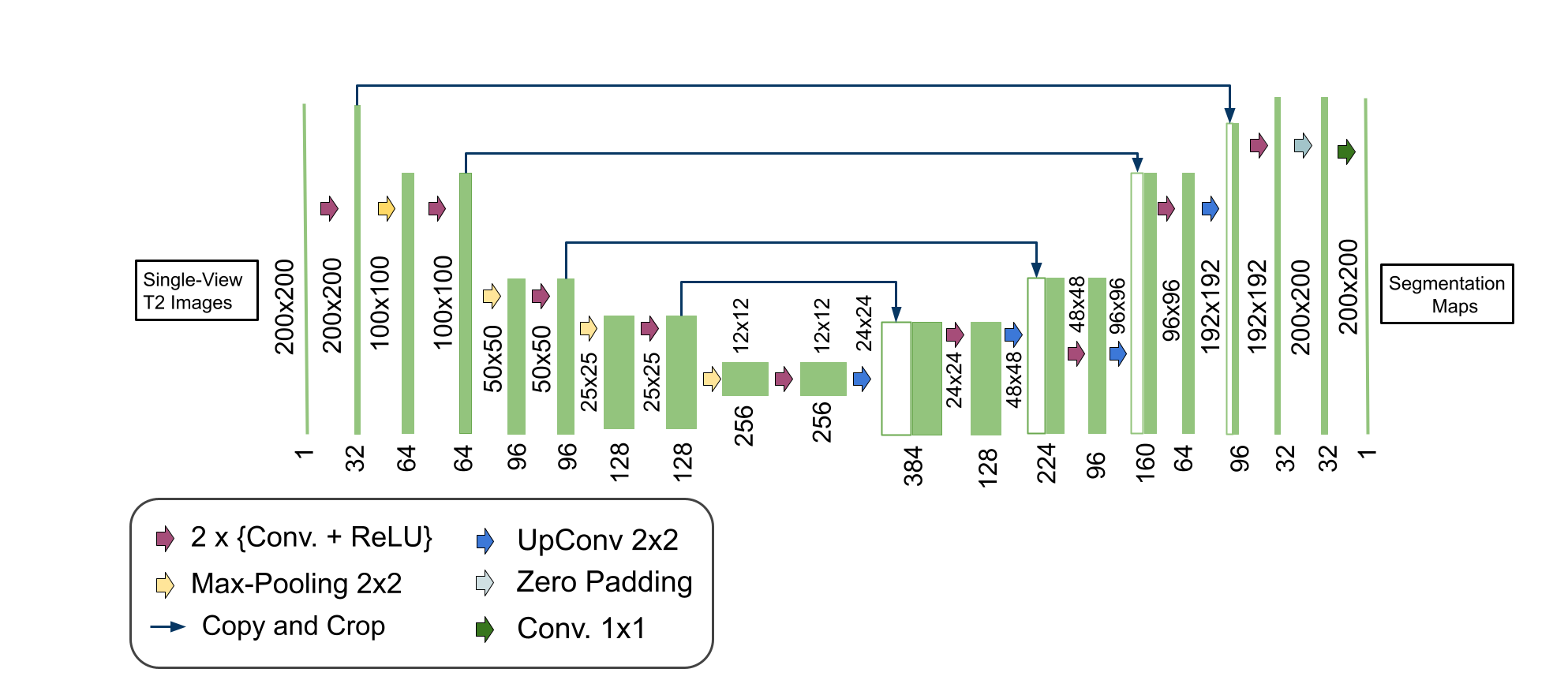
**

**Fig. S2** Architecture of the 2D Convolutional neural network. This u-net structure with an encoding and a decoding part is used for axial and coronal models, respectively. It deals with a T2-weighted image slice as input and provides a 2D segmentation map as output.


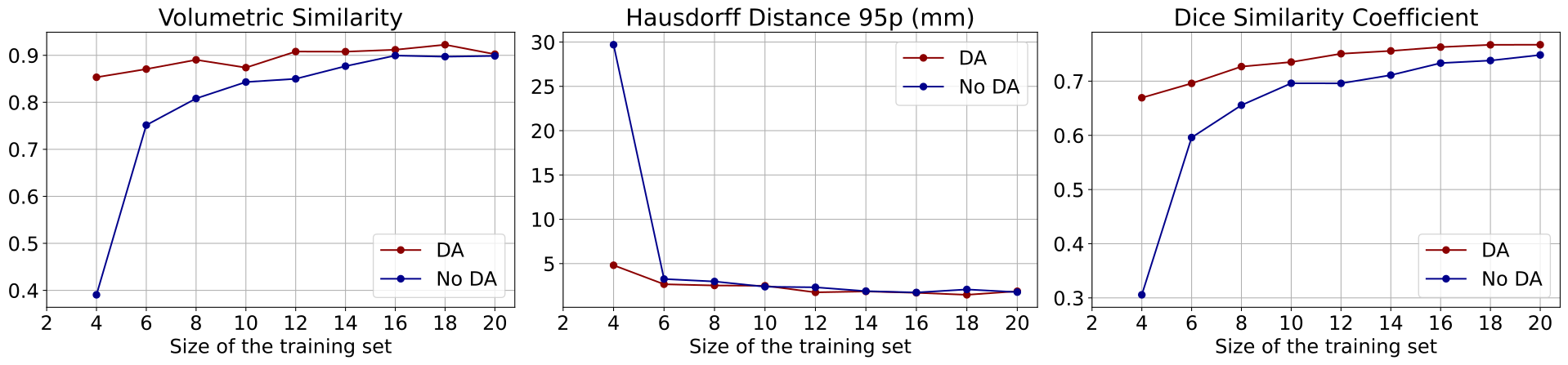


**Fig. S3** Effectiveness of data augmentation (DA) with a variable amount of training images. For these experiments, DA models were trained for 30 epochs while models without DA were trained for 50 epochs. Expanding the training of models without DA from 50 to 60 epochs did not enhance the accuracy. DA models outperform non-DA models with equal numbers of scans for training. Training a model with twelve scans and DA is as effective as training a model with 20 scans without DA. Thus, DA can reduce the manual segmentation work.

**
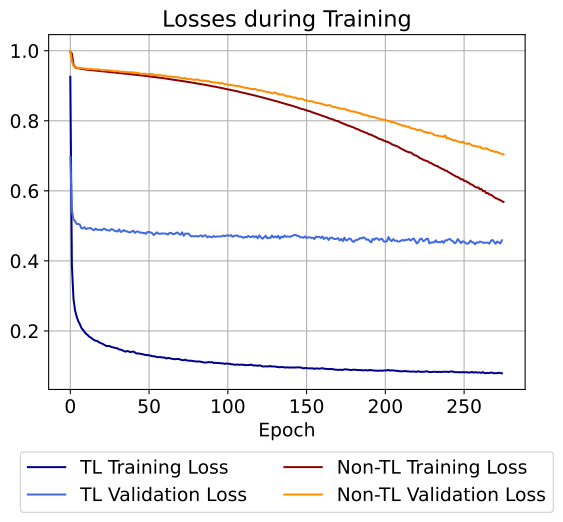
**

**Fig. S4** Training and validation loss of one transfer learning (TL) and one non-transfer learning (Non-TL) model which were trained with 16 images and tested on four scans of the training set, respectively. For illustration, the TL model was trained for 275 epochs although we chose a number of 30 epochs in our experiments. Transfer learning is faster and presents lower losses in the whole range pointing to a more distinct differentiation of claustrum and non-claustrum voxels in MR images.

**
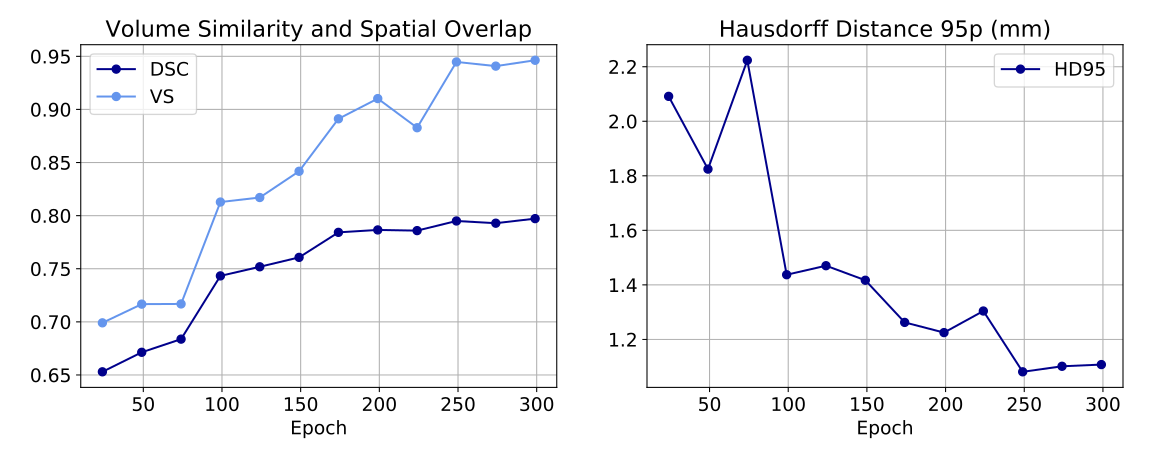
**

**Fig. S5** To choose the optimal number of epochs for non-transfer learning, we trained an axial and a coronal model with 16 images and tested it on four scans of the training set. For reasons of clearness, we only plot the result of one coronal model for every 25th epoch. Volumetric similarity (VS), Dice similarity coefficient (DSC) and Hausdorff distance (HD95) stabilize between 250 and 300 epochs. Thus, we chose 275 epochs for non-transfer learning. (VS=volumetric similarity, HD95=95^th^ percentile of Hausdorff Distance, DSC=Dice similarity coefficient)

**
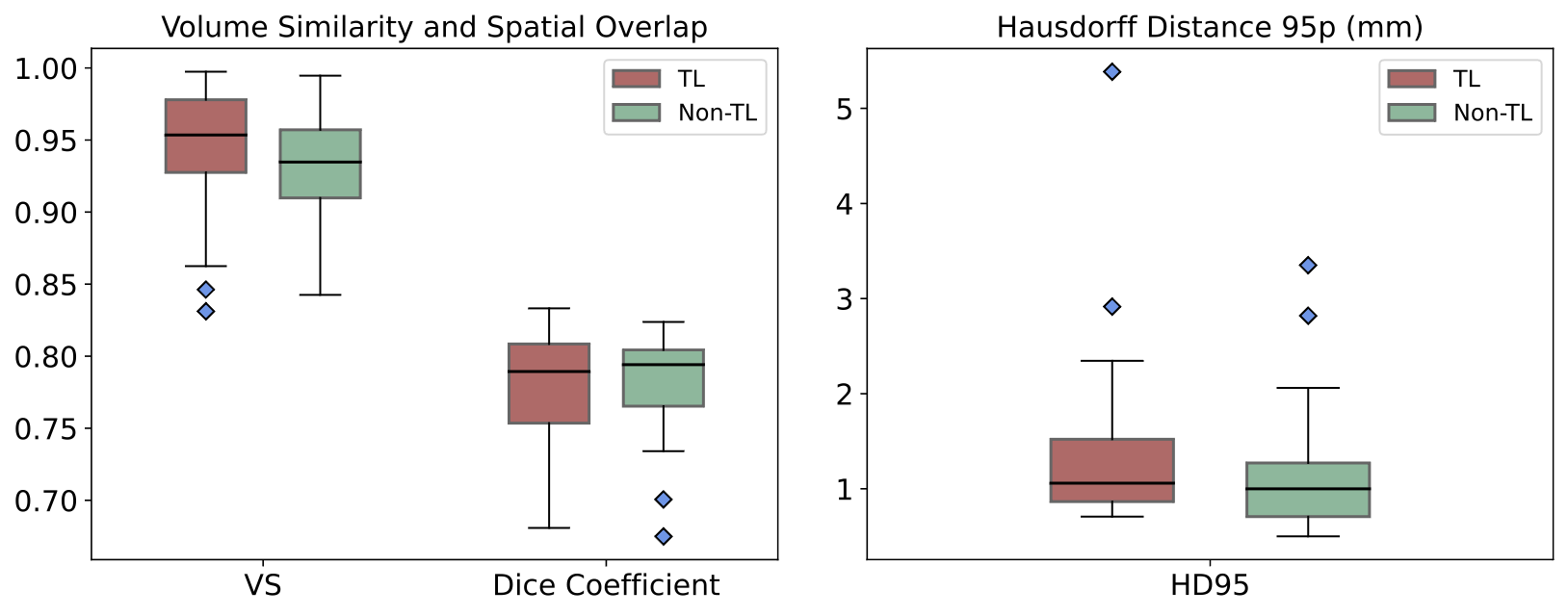
**

**Fig. S6** Comparison of two methods, transfer learning (TL, trained for 30 epochs) and non-transfer learning (Non-TL, trained for 275 epochs), in the internal five-fold cross-validation on the training set. The models were trained with 16 scans and tested with four scans in each fold. TL showed superior VS, inferior HD95 and comparable DSC like non-TL with a Wilcoxon signed-rank test. (VS=volumetric similarity, HD95=95^th^ percentile of Hausdorff Distance, DSC=Dice similarity coefficient)

**Table S2** Performance comparison of the models with and without transfer learning (TL). TL achieves significantly higher VS, lower HD95 and comparable DSC in a Wilcoxon signed-rank test. TL is much faster than non-TL. ↓ indicates that a smaller value represents better performance. (VS=volumetric similarity, HD95=95th percentile of Hausdorff Distance, DSC=Dice similarity coefficient, IQR=interquartile range)

| Metrics | VS (%)  Median, [IQR] | HD95(mm)↓  Median, [IQR] | DSC (%)  Median, [IQR] | Training time (hours), [number of epochs] |
| --- | --- | --- | --- | --- |
| Non-TL | 93.5, [91.0, 95.7] | 1.00, [0.71, 1.27] | 79.4, [76.5, 80.4] | 17.5, [275] |
| With TL | 95.3, [92.8, 97.8] | 1.06, [0.87, 1.52] | 78.9, [75.4, 80.8] | 2.0, [30] |
| p-value | **0.050** | **0.016** | 0.452 | -- |


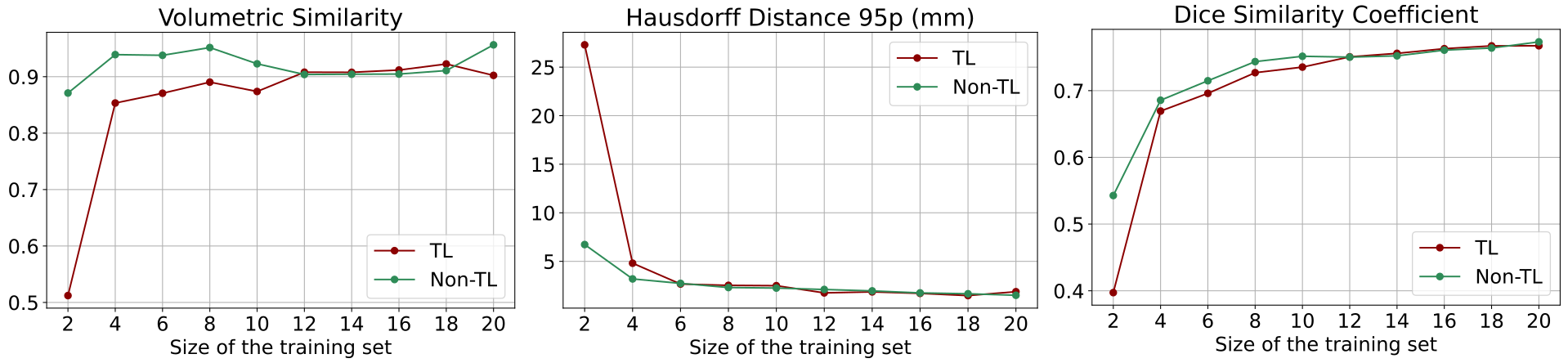


**Fig. S7** Needed training data for transfer learning (TL) in comparison with non-transfer learning (non-TL). With a small training set, non-TL achieves slightly higher accuracy than TL. Starting from twelve images in the training set, both methods show comparable high-level performance. For these experiments, TL models were trained for 30 epochs while non-TL models were trained for 275 epochs.

**
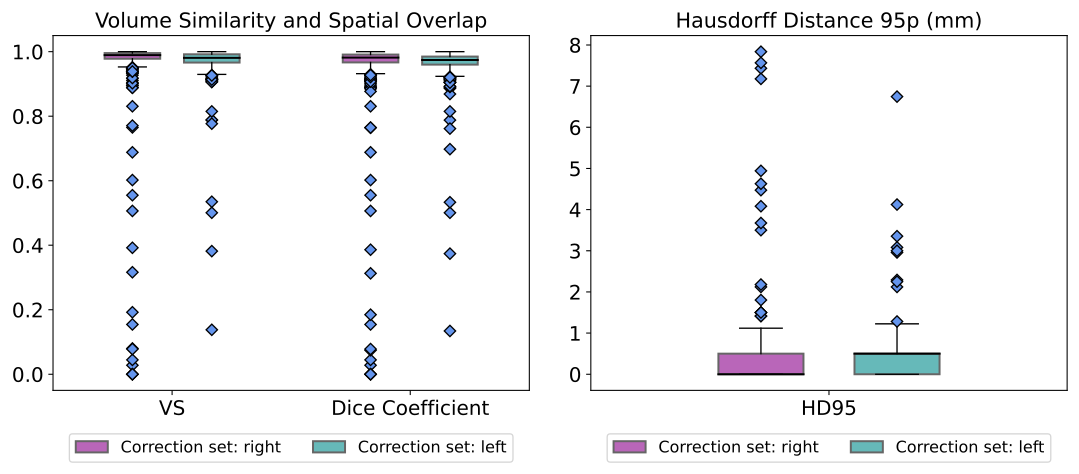
**

**Fig. S8** Box plots showing the agreement between automated segmentation of the combined model and subsequent manual correction of it separated for right and left claustrum. Most of the automated segmentations reach very high scores. (VS=volumetric similarity, HD95=95^th^ percentile of the Hausdorff Distance, DSC=Dice similarity coefficient)

**Table S3** Comparison of automated and subsequently corrected segmentation regarding right and left claustrum of the correction set comprising 528 scans. The combined model detects the right claustrum slightly better than the left although there are more outliers regarding the right claustrum (see Figure S7). ↓ indicates that a smaller value represents better performance. (VS=volumetric similarity, HD95=95th percentile of Hausdorff Distance, DSC=Dice similarity coefficient, IQR=interquartile range)

| Metrics | VS (%)  Median, [IQR] | HD95(mm)↓  Median, [IQR] | DSC (%)  Median, [IQR] |
| --- | --- | --- | --- |
| Right claustrum | 98.9, [97.8, 99.6] | 0.00, [0.00, 0.50] | 98.2, [96.7, 99.1] |
| Left claustrum | 98.1, [96.6, 99.2] | 0.50, [0.00, 0.50] | 97.4, [96.6, 99.2] |
| p-value | **<0.0001** | -- | **<0.0001** |

**
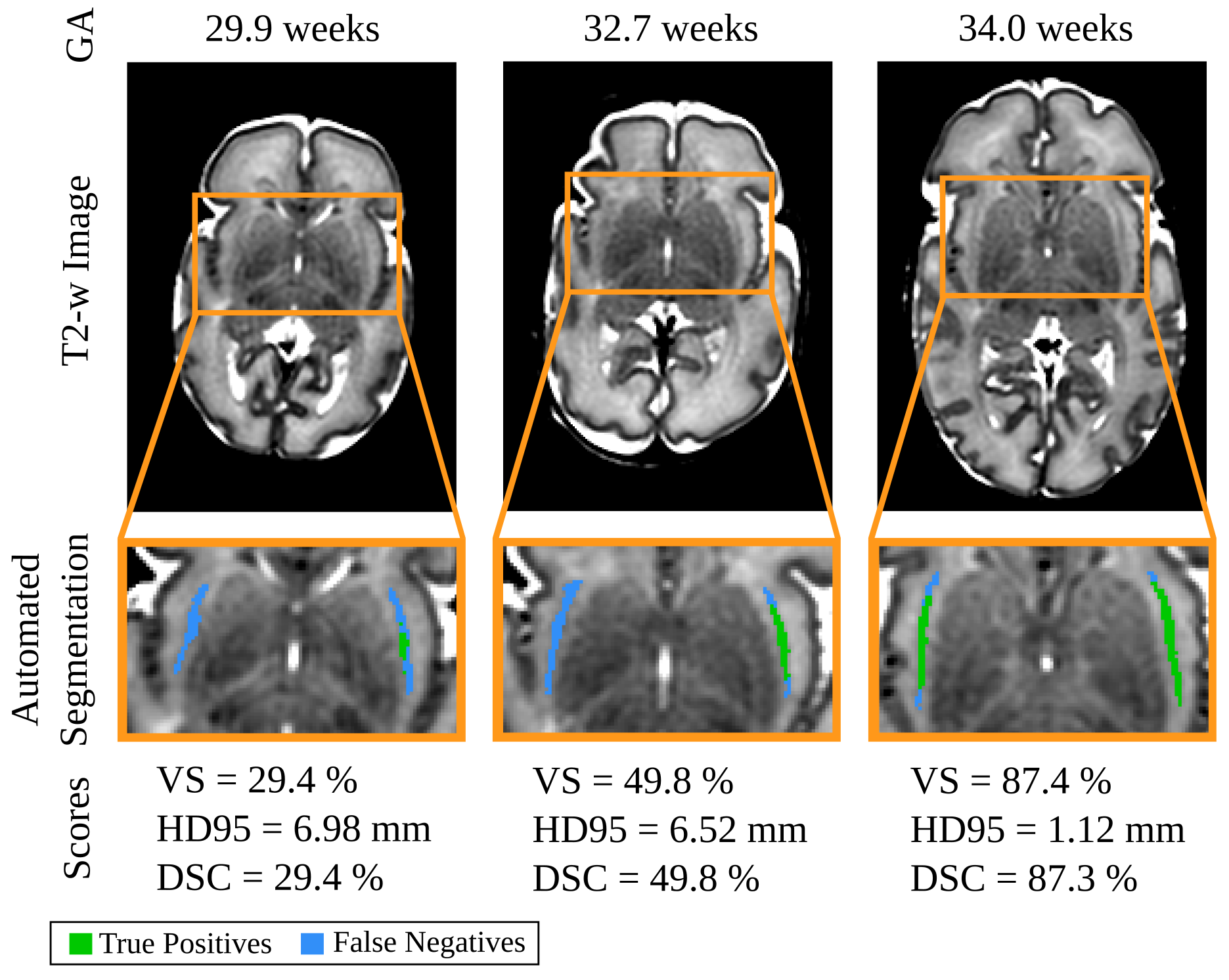
**

**Fig. S9** Segmentation results of three cases which were manually corrected after prediction. In the automated segmentation masks, the green pixels represent true positives and the blue ones represent false negatives. False positives are not present in these slices. Examples are sorted according to the gestational age of the scanned subjects (GA=gestational age, VS=volumetric similarity, HD95=95th percentile of the Hausdorff Distance, DSC=Dice similarity coefficient)


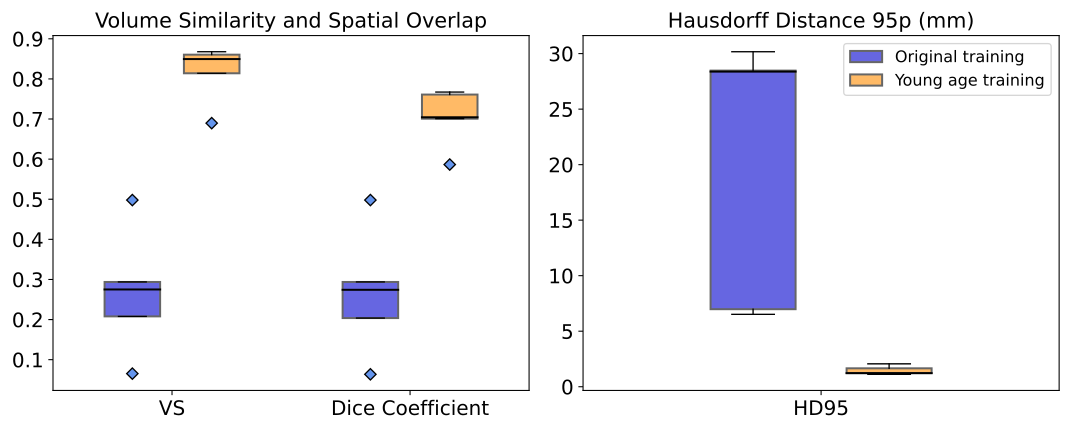


**Fig. S10** Comparison of two training settings (Young age training = age-stratified vs Original training = non-age-stratified). Subsequently, the combined axial and coronal view models were tested on the five young subjects (scan age range: 29.3-32.7 gestational weeks) with the lowest Dice similarity coefficient by the model trained with the original training set.

**Table S4** Comparison of two training settings (Young age training = age-stratified vs Original training = non-age-stratified). The combined axial and coronal view models were tested on the original test set of ten subjects (scan age range: 38.7-42.3 gestational weeks). We found that the approach trained with two younger subjects significantly outperformed the original training set regarding the volumetric similarity. ↓ indicates that a smaller value represents better performance. (VS=volumetric similarity, HD95=95th percentile of Hausdorff Distance, DSC=Dice similarity coefficient, IQR=interquartile range)

| Metrics | VS (%)  Median, [IQR] | HD95(mm)↓  Median, [IQR] | DSC (%)  Median, [IQR] |
| --- | --- | --- | --- |
| Original training set | 95.9, [95.4, 97.2] | 1.12, [1,12, 1.34] | 80.0, [78.4, 81.2] |
| Including young age scans | 97.6, [95.7, 98.4] | 1.12, [1.02, 1.20] | 79.9, [77.8, 80.9] |
| p-value | **0.009** | 0.091 | 0.508 |

**Claustrum segmentation protocol for neonatal brain MRI**

T2-weighted images are provided by the developing Human Connectome Project (<http://www.developingconnectome.org/project/>).

1. Find a slice in axial sight where you can easily recognize the claustrum. Then adjust the image contrast to optimize the differentiation of the claustrum in comparison to surrounding white matter.


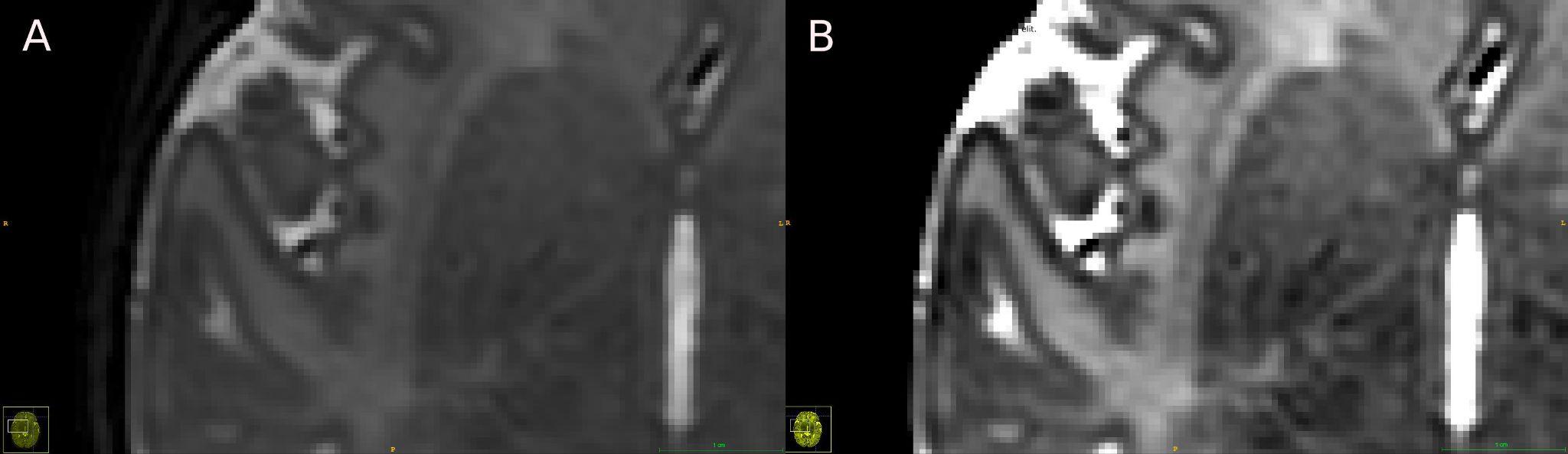


1. Select the axial plane and zoom in. The favored extract captures the claustrum and adjacent anatomical structures like the putamen and the insula for orientation. Single voxels should be identifiable without struggle.
2. To start with actual segmentation, focus on the claustrum of interest. In this protocol, all integrated figures show sections with the right claustrum. Choose a slice with visible claustrum with long continuous appearance in anterior-posterior dimension and trace it with a selected label. Avoid marking any voxel which is part of the insular cortex or the basal ganglia. If the claustrum seems to be directly next to them try to leave a small distance between segmentation and neighboring structure.


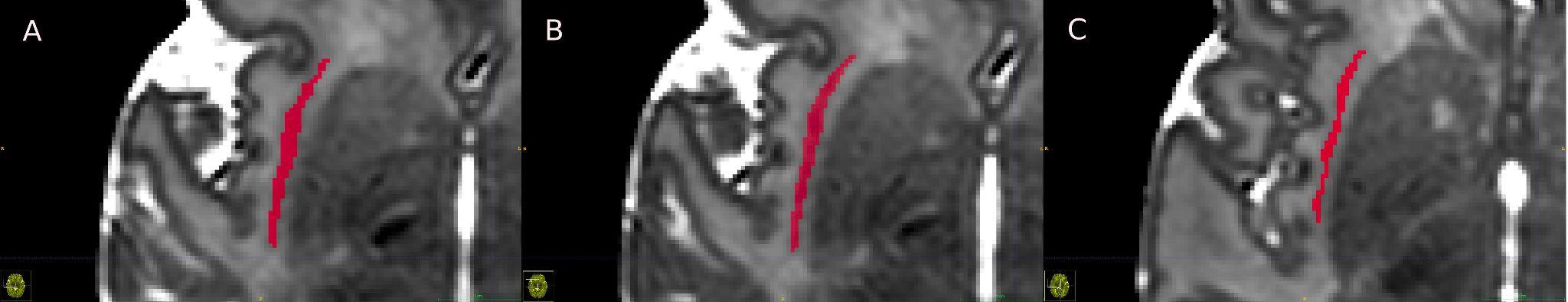


1. Continue with the slice below and go on until you can not surely define the measure of the claustrum anymore. In general, do not include rays of gray matter which sometimes emerge from the claustrum. Only incorporate voxels at the same level like the residual claustrum surface.


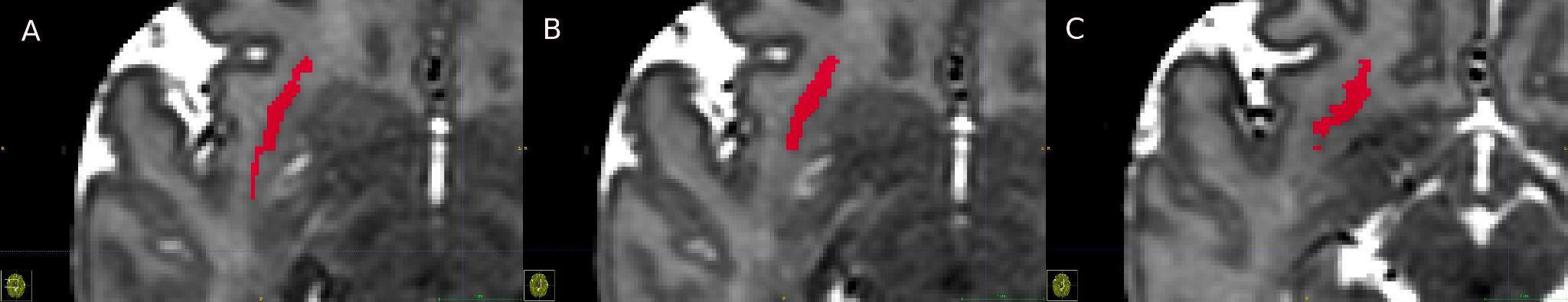


1. Go back to your starting slice and repeat the previous step but in the dorsal direction. In this direction, the visible claustrum is often separated in several parts.


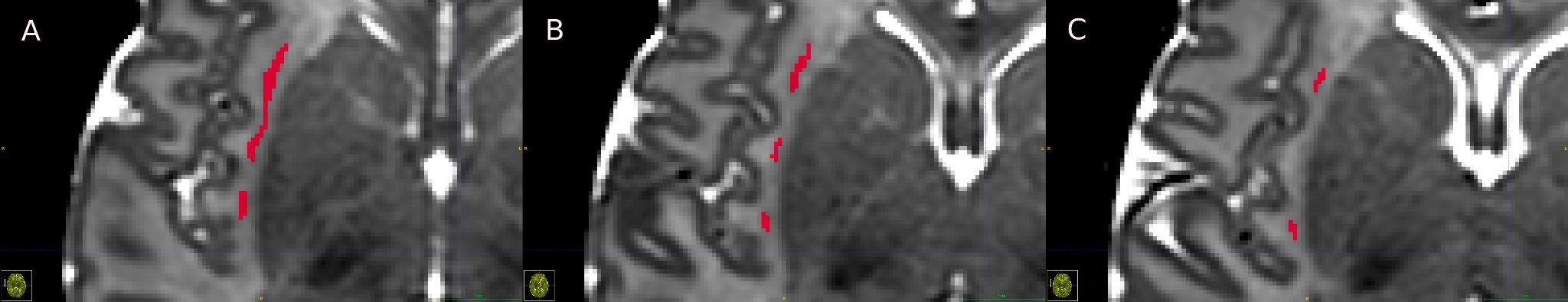


1. Afterward, switch to the coronal plane and again start with a central slice and go backward slice per slice, then forward. Supplement the segmentation especially the ventral part of the claustrum which expands under the putamen. It is easier to assess this area in coronal than in axial view. Correct the tracing if necessary by using the "clear label“. Therefore, it is helpful to modulate the opacity of the label.


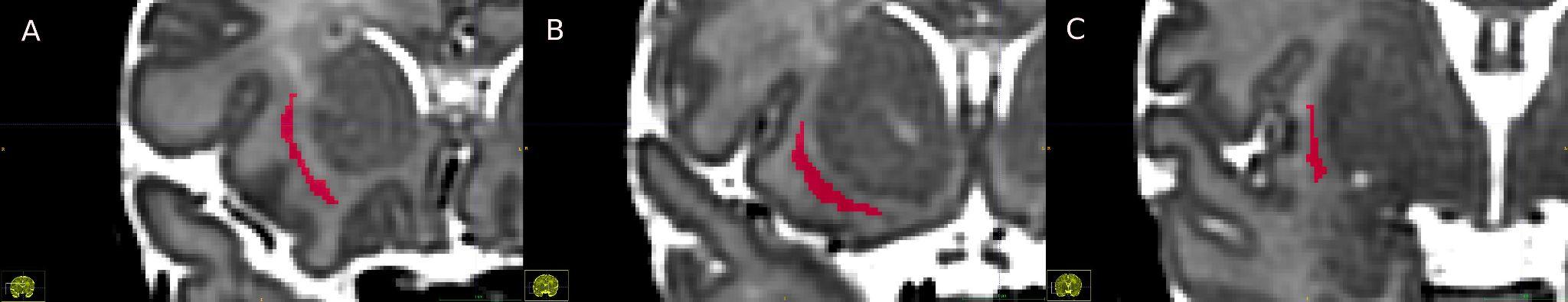


1. Return to the axial plane and correct details where needed to enhance reliability. Again, mind a small distance between segmented claustrum and other structures.
2. To trace the second claustrum, repeat steps three to seven with another label if requested.
